# Extended use of point-of-care technology versus usual care for in-home assessment by acute community nurses in older adults with signs of potential acute respiratory disease: an open-label randomised controlled trial protocol

**DOI:** 10.1186/s12877-024-04774-z

**Published:** 2024-02-16

**Authors:** Siri Aas Smedemark, Christian B. Laursen, Dorte Ejg Jarbøl, Flemming S. Rosenvinge, Karen Andersen-Ranberg

**Affiliations:** 1 Department of Geriatric Medicine, Geriatric Research Unit, Odense University Hospital, Odense, Denmark; 2 Department of Clinical Research, University of Southern Denmark, Odense, Denmark; 3 Department of Respiratory Medicine, Odense Respiratory Research Unit, Odense University Hospital, Odense, Denmark; 4 Department of Public Health, Research Unit of General Practice, University of Southern Denmark, Odense, Denmark; 5 Department of Clinical Microbiology, Odense University Hospital, Odense, Denmark

**Keywords:** Point-of-care technology, Geriatric Assessment, Acute community nurse, Focused lung ultrasound

## Abstract

**Background:**

Due to ageing-related physiological changes, diagnosing older adults is challenging. Delayed disease recognition may lead to adverse health outcomes and increased hospitalisation, necessitating the development of new initiatives for timely diagnosis and treatment of older adults. Point-of-care technology, such as focused lung ultrasound scan and bedside analysis of blood samples (leucocytes with differential count, electrolytes, and creatinine) conducted in the patients’ home, may support clinical decision-making, and potentially reduce acute hospital admissions.

We present the protocol for a randomized controlled trial, which aims at assessing the effect of focused lung ultrasound scan and bedside blood analysis during in-home assessments among older adults with signs of potential acute respiratory disease on hospital admissions.

**Method:**

We will use a parallel open-label, individually randomised controlled trial design in an acute community healthcare setting. The trial will initiate on October 2022 and is expected to end one year later. The study population will include older adults (65 + year), with at least one of the following inclusion criteria: Cough, dyspnoea, fever, fall, or rapid functional decline. Expected study sample will comprise 632 participants. Participants in the control group will receive usual care, while the intervention group will undergo extended point-of-care technology (focused lung ultrasound scan and bedside venous blood analysis), in addition to usual care.

The primary outcome is acute hospital admission within 30 days follow-up. Secondary outcomes include readmissions, mortality, length of hospital stay, hospital-free days, complications during hospital admission, treatment initiations or changes, functional level, re-referrals to the acute community healthcare service, and contacts to the primary care physician. A tertiary outcome is the diagnostic accuracy of Acute Community Nurses for conducting focused lung ultrasound compared with a specialist. Outcomes will be analysed as intention-to-treat.

**Discussion:**

To our knowledge, this is the first randomised controlled trial examining the effect of extended use of point-of-care technology conducted in an in-home setting. We expect that the results may contribute to the development of new interventions aiming to improve timely diagnostics, treatment decisions, and reduce acute hospital admissions.

**Trial Registration:**

www.clinicaltrials.org NCT05546073 (Date of registration: September 19th, 2022).

**Supplementary Information:**

The online version contains supplementary material available at 10.1186/s12877-024-04774-z.

## Background

The population of adults aged 65 years and above is increasing, posing challenges to high-income countries’ primary and secondary healthcare systems [1, 2]. Of particular concern is the high rate of acute hospital admissions and in-hospital complications [3–9]. Recognising that timely treatment may prevent the progression to severe disease, it is imperative to identify and diagnose acute disease in older adults (65 + years of age) as soon as possible to avoid acute hospitalisation and functional decline [9–11]. Diagnosing older adults can be challenging due to ageing-related physiological changes [12, 13]. Moreover, many older adults do not present with typical symptoms; for example, coughing is a less prominent symptom of pneumonia [14]. Instead, older adults often exhibit atypical symptoms, such as delirium, falls, and rapid decline in physical functions, which are common proxy symptoms for infections [15].

Worldwide, lower respiratory tract infections rank among the most common reasons for hospitalization among older adults [16–18]. Given the challenges of diagnosing older adults and preventing acute hospital admission, a comprehensive approach is needed. This requires clinical examination, assessment of vital signs, and use of diagnostic tools, as well as biochemical results [12].

Point-of-care technology (POCT) allows tests or examinations to be carried out bedside, i.e., in the patient’s own dwelling, providing rapid answer in less than 20 min, depending on the instruments used [19, 20]. In primary care, commonly used POCT includes tests for glucose, International Normalized Ratio (INR), and C-reactive protein [21]. In recent years, new POCT for blood-analyses, such as leucocytes, creatinine, electrolytes, have been developed; however, they are not widely used in primary care [22]. As POCT may be used in a home-setting, it seems to be well-suited for older adults as diagnostics tools may support timely clinical decision making [21].

Point-of-care ultrasound (POCUS) has become a diagnostic tool for acute respiratory diseases over the last couple of decades. Studies have shown, that in the hands of a trained health-professional, focused lung ultrasound (FLUS), is an accurate tool for diagnosing pulmonary oedema, pneumothorax, pleural effusion, and pneumonia in emergency departments and intensive care units. [23–25]. However, there is no knowledge about its use and effects on the diagnostic process during in-home assessments. Only few studies have investigated FLUS as a diagnostic tool for pneumonia in primary care, but these studies were not representative for the geriatric population [26, 27].

The objective of this trial is to investigate whether extended POCT, including FLUS and bedside blood analysis, during in-home assessments among older adults with signs of potential acute respiratory disease, reduces hospital admission. Our primary hypothesis is that implementation of extended POCT in this context will result in a reduction in hospital admissions within 30 days compared to individuals receiving usual care, constituting our primary outcome. Additionally, we anticipate that the extended POCT will improve the accuracy and timeliness of bedside diagnostics, leading to enhanced clinical decision-making by primary care physician – reflecting our secondary outcomes and tertiary outcomes. Finally, we hypothesize that the intervention will lead to earlier identification of conditions and diseases, subsequently influencing factors such as hospital length of stay, amount of home care, and functional status during the 30 days follow up. This early diagnosis is expected to prevent functional decline, aligning with our secondary outcomes.

## Methods

This protocol paper is reported in line with SPIRIT statements [28], and the trial was registered at www.clinicaltrials.gov (Trial registration: NCT05546073, date of registration: September 19th, 2022). Any protocol amendments or modifications will be registered at www.clinicaltrial.gov.

### Study design

The trial is an open-label individually randomised controlled trial that investigates whether extended use of POCT for in-home assessment among older adults aged 65 and older reduces the rate of acute hospital admissions compared with usual care. The trial will use a parallel group design with a 1:1 allocation ratio, and a superiority design.

### Study setting

The trial is conducted in Kolding Municipality, Denmark, covering an area of 604.5 km^2^ with a population of 93,161 people, of whom 18,453 are aged 65 years or older [29].

In 2018, it was enforced to all Danish municipalities to establish an Acute Community-based Health Care Service (ACHCS) to enhance the timely recognition of acute disease [30]. While municipalities had the freedom to organise the ACHCS, most introduced an outgoing function from community healthcare centres, operated by Acute community nurses (ACNs).

The ACHCS in Kolding Municipality has a stationary function and an outgoing function, with ACNs dedicated to the outgoing function. The ACNs conduct in-home assessments primarily for older vulnerable citizens with acute signs of health deterioration. Approximately 5 – 8 patients are referred daily for in-home assessment due to suspected emerging acute disease. Referrals are mainly made by PCPs, but hospital physicians and home care personnel also have the capability to make referrals. If home care personnel refer patients, the PCP will be notified and responsible for diagnosis and treatment.

The assessment of acutely ill patients includes a clinical assessment, objective vital status parameters (oxygen level, respiration frequency, pulse, blood pressure, temperature), and, when appropriate, on-site POCT for biochemical analyses, e.g., CRP and haemoglobin. All results are communicated to the PCP or the hospital physician, providing them with clinical, objective information that qualifies diagnosis and medical decision-making.

### Study participants

Participants eligible for this study are people aged 65 years or older, referred to the ACHCS in Kolding Municipality for an in-home assessment.

The participants must have at least one of following symptoms of respiratory disease: cough, dyspnoea, fever, fall, or recent functional decline, defined as subjective (not able to perform normal daily activities as usual) or objective functional decline (increased need of home care). Falls and functional decline are not directly symptoms of respiratory disease, but are known as vague and atypical disease presentation; which is why we included them as signs of potential respiratory disease [31, 32].

Participants with known moderate to severe cognitive impairment are excluded from the study due to Danish legislation and the Regional Committees on Health Research Ethics for Southern Denmark.

### Recruitment and enrolment

Patients referred to the ACHCS are visited by an ACN in their own dwelling, which may include a private house, care-home, or rehabilitation centre. If the patient fulfils the inclusion criteria, the ACN provides comprehensive information about the study both orally and in writing. Subsequently, the patient is given the opportunity to sign the informed consent document. Additional files 1 and 2 contain details pertaining to the informed consent process and written participant information. These documents have received approval from the Regional Committees on Health Research Ethics for Southern Denmark.

### Randomization, allocation, and implementation

**Fig. 1:**
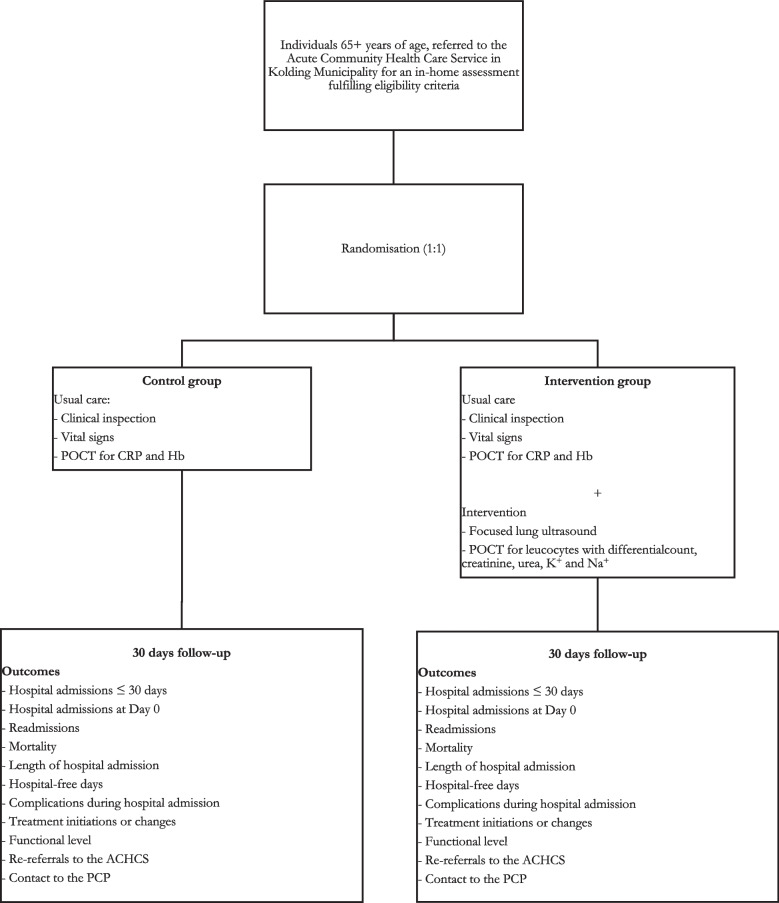
Study Flow Chart. ACHCS, Acute Community Health Care Service; POCT, Point-of Care Technology; CRP, C-reactive Protein; Hb, Hemoglobin; PCP, Primary Health Care Physician

The allocation sequence of randomisation numbers is generated using an online random number service, carried out by a data manager from Open Patient Data Exploratory Network (OPEN) [33] and the primary investigator, Siri Aas Smedemark. Randomisation numbers are paired with the REDCap® database (version: REDCap 9.1.15—© 2020 Vanderbilt University) developed for this trial by the primary investigator [34, 35]. Upon obtaining the participant’s signed informed consent, the ACN will register the participant in the trials database. The database employs a random allocation system, assigning a unique computerized randomization number to each participant. All parties involved, including participants, ACNs, and PCPs are aware of group assignment. Figure 1 shows the study flow.

### Intervention and control

In the control group, the ACN will carry out usual care, which involves a clinical inspection, measurements of vital signs (respiratory rate(breaths/min), saturation (%), blood pressure (mmHg), heart rate (beats/min), body temperature (C^0^), and Glasgow Coma Scale [36]), and POCT for CRP and haemoglobin.

The intervention group will receive extended POCT in addition to usual care. Extended POCT includes a FLUS and biochemical blood analyses. Biochemical blood analyses include sodium, potassium, creatinine, urea, and leucocytes with differential count.

In both control and intervention groups, the ACN communicates the examination results to the participants’ PCP through telephone communication and electronic message (EdiFact) for written documentation. The communication to the PCP is part of usual care and is carried out in both groups.

Preceding to commencement of the study, all ACNs undergo comprehensive training in FLUS, technical procedure of POCT biochemical blood-analysis, as well as training in research methods with a specific emphasis on study procedures. This training is designed to enhance their adherence to and compliance with the randomization procedure and protocol.

### FLUS

The training program for ACNs in FLUS encompasses four integral modules: The initial module involves online training (Module 1). This entails theoretical instruction in sonographic physics, knobology, and sonographic findings related to FLUS. Module 1 is delivered through an e-learn platform (Plan2Learn ApD, Viby, Denmark) with a validated theoretical test in FLUS [37]. Successful completion is a prerequisite for advancing to Module 2—Immersive Virtual Reality-FLUS training. Module 2 is developed by Vita-Sim (VitaSim ApS, Odense, Denmark) and Jonas Dragsbæk Larsen (J.D.L.) (Department of Radiology, Odense University Hospital) [38]. Following Module 2, ACNs proceed to Module 3 hands-on FLUS-training, provided by the primary investigator. Subsequent to this, ACNs engage in supervised daily FLUS-training for approximately two months (Module 4).

Certification of ACNs in FLUS is required upon their evaluation through the validated objective structured assessment of technical skills tool for point-of-care lung ultrasound imaging (LUS-OSAUS), conducted at intermediate level [39].

FLUS is performed using Lumify® C5-2 Curved Array Transducer (Philips Medical Systems, Bothell, WA), lung preset, with a bandwidth of 5–2 MHz and scan depth up to 30 cm.

A standardized protocol, previously validated in patients with acute respiratory symptoms, guide FLUS diagnostic criteria during the trial [24, 40]. These criteria align with international recommendations [41–43]. ACNs document conclusive findings (See Additional file 3). All FLUS examinations will be stored digitally, and undergo blinded assessment by a specialist throughout the RCT. The ACNs will receive feedback on their FLUS during the RCT once a week by primary investigator.

In instances where ACNs encounter challenges in interpreting FLUS findings during in-home assessments, the option of tele-ultrasound through Cisco Webex® (Cisco system, Inc., San Jose, U.S.A) is available. This functionality facilitates online video calls, enabling ACNs to seek guidance from the primary investigator. Utilization of Webex® is recorded, along with reasons for its application during the study period.

### Venous blood samples

Medical laboratory technologists from Hospital Lillebaelt, Kolding Hospital, will train ACNs in correct venous blood sample techniques.

POCT on blood samples for creatinine and electrolytes involves venous blood samples collected in lithium/heparin vacutainers. The samples are immediately analysed using the i-STAT® (Abbott, Inc., NJ, U.S.A.) with the CHEM8 cassette.

For POCT blood analysis of leucocyte differential count, venous blood samples are collected in EDTA-vacutainers and analysed immediately on the HemoCue® WBF DIFF System (HemoCue AB, Ängelholm, Sweden) with dedicated micro-cuvettes.

Additionally, POCT blood-analysis for CRP is conducted using blood samples obtained by finger-prick and analysed on the QuickRead go® instrument (Orion Diagnostica, Oy).

Technical handling of all POCT instruments will be demonstrated by the POCT-companies.

Routine venous blood samples will be collected monthly to validate the POCT equipment. The samples will be analysed at the Department of Biochemistry and Immunology, Hospital Lillebaelt, Kolding Hospital, within 6 h. To maintain sample integrity during transport, the samples will stored in a controlled cabinet, maintaining a stable temperature of 20 °C.

### Research training session

All ACNs will receive a research training session on “How to conduct research”, with specific focus on informing eligible participants about the trial, and the importance of adherence to protocol. The primary investigator is responsible for conducting the research training session.

### Outcomes

The primary outcome of this trial is the rate of hospital admission within 30-days follow-up. Secondary outcomes, along with their definitions, are listed in Table 1. The tertiary outcome involves evaluating ACNs diagnostic accuracy in conducting FLUS compared with a FLUS-specialist. A FLUS specialist will perform blinded assessment of all FLUS video clips. Outcome measures will include sensitivity and specificity, PPV and NPV, with a Bland-Altmann analysis. The results of the tertiary outcome will be reported in a separate paper.

**Table 1:** Primary, secondary, and tertiary outcomes

	**Outcome**	**Outcome measure**
***Primary outcome***		
Outcome 1	Hospital admissions ≤ 30 days	Proportion of hospital admissions within 30 days follow-up (Day 0^a^ to Day 30)
***Secondary outcomes***		
Outcome 2	Hospital admissions at Day 0^a^	Proportion of hospital admission at Day 0^a^
Outcome 3	Readmission ≤ 30 days	Proportion of readmissions within 30 days follow-up (Day 1 to Day 30)
Outcome 4	Mortality ≤ 30 days	Number of deaths within 30 days follow-up (Day 0^a^ to Day 30)
Outcome 5	Length of hospital admission	Number of days admitted
Outcome 6	Hospital-free days ≤ 30 days	Number of days alive that is spend outside of an acute-care hospital, long-term acute-care hospital or in an emergency department. Days spent wholly or in part under “observation” status counts as hospital days [44]
Outcome 7	Complications during hospital admission	Number of complications during admission, registered in the electronic patient journal
Outcome 8	Treatment initiations or changes at Day 0^a^	Number of treatment initiations or changes at Day 0^a^
Outcome 9	Treatment initiation or changes ≤ 30 day	Number of treatment initiations or changes within 30 days follow-up (Day 1 to Day 30)
Outcome 10	Functional level	Proxy assessment of functional level: - Change in amount of home care within 30 days follow-up - Number of changes of dwelling
Outcome 11	Re-referrals to the ACHCS	Number of re-referrals to the ACHCS
Outcome 12	Contact to the PCP ≤ 30 days	Number of contacts to the PCP within 30 days follow-up (Day 0^a^ to Day 30)
***Tertiary outcomes***		
Outcome 14	ACNs diagnostic accuracy for conducting FLUS compared with a FLUS-specialist	ACNs conclusions are index test and FLUS-specialist conclusions used as reference test

*ACNs* Acute community Nurses, *ACHCS* Acute Community Health Care Service, *FLUS* Focused Lung Ultrasound, *PCP* Primary Care Physician

^a^Day 0: Defined by Day of inclusion

### Data collection

During the in-home assessment participants will undergo a systematic screening and examination program, which include registration of age, gender, alcohol consumption, smoking, BMI, nationality, comorbidity, polypharmacy, place of assessment, symptoms of infection, days with symptoms, Clinical Frailty Scale [45], vital signs (blood pressure, pulse, oxygen saturation, respiratory frequency, and ear temperature), and POCT for analyses of CRP and haemoglobin.

In addition, the intervention group will undergo the extended POCT examinations (FLUS and biochemistry on blood analysis).

Outcomes for the 30 days-follow up period are collected from electronic databases. Table 2 provides an overview of the data-sources and corresponding time-points for each outcome.

**Table 2:** Time point and data-sources for primary and secondary outcomes

	**Day 0**^**a**^			**Follow up: 30 days**			
	**During in-home assessment**	**ESCR**	**Danish health registries**	**ESCR**	**EPJ**	**FMK**	**Danish health registries**
**Primary outcome**							
Hospital admission	x		x		x		x
**Secondary outcomes**							
Readmission					x		x
Death					x		x
Hospital-free days							x
Length of hospital stay					x		x
Complications during hospital stay					x		
Treatment initiations	x					x	x
Treatment changes						x	x
Amount of home care		x		x			
Re-referral to ACHCS				x			
Contact to PCP			x				x
*Data collector*	*ACN*	*Nexus administrator*	*Primary investigator*	*Nexus administrator and ACNs*	*Primary investigator*	*Primary investigator and ACNs*	*Primary investigator*

*ESCR* Electronic social care record, *EPJ* Electronic patient journal, *FMK* Medical chart (Fælles medicinkort)

^a^Day 0: Defined by Day of inclusion

### User perspectives

User perspectives on the intervention will be explored during the RCT. PCPs, ACNs, and participants will be invited to focus group interviews. Qualitative data from the user perspectives will be reported in a separate article.

### Advisory board

An advisory board, comprised of PCPs, ACNs, hospital physicians, and researchers, has been formed. The Advisory Board actively participated in the development of the working algorithm and provides support for fostering collaboration between health care providers in both the primary and secondary sector throughout the trial.

### Data sources

We will apply for access to external data sources to assess co-morbidity, disabilities, utilization of primary care and health care services, death, and medical history.

The Danish Health Data Authority is responsible for the national health registers containing health data related to all Danish citizens [46, 47]. A unique personal identification number for all people living in Denmark makes it possible to link data across national health registers. Approval granted August 2023.

The Danish Health Data Authority will be applied to get access to the Danish National Patient Registry, the Cause of Death Register, The Register of Pharmaceutical Sales, and the Danish National Health Service Register. The registers contain information about diagnoses, treatments, deaths, and health care utilization, and are needed to access information on comorbidity, time of death, medical history, treatment initiations or changes, and number of consultations at PCPs.

The Medical Chart (FMK: Fælles MedicinKort), will be applied to access register polypharmacy and type of medications. Data on amount and type of medication will be obtained at day 0 by the primary investigator. Treatment initiations or changes within 30 days follow-up (Day 1 to day 30) will be obtained by the primary investigator on Day 30. Approval granted October 2022.

The Municipal journal record, Electronic Social Care Record (ESCR), registers home care, and data will be obtained at day 1 and at day 30 to investigate possible changes as a proxy of changes in functional level. Approval granted October 2022.

The hospital journal record, EPJ will be accessed by the primary investigator to register admissions, reason for admission, and complications during admission. EPJ will be accessed at day 30 after the acute visit. Approval granted October 2022.

### Data management

#### Data documentation

The project is registered with the Research & Innovation Organization (RIO), University of Southern Denmark, record of data processing activities, (Project identification number: 11.404). Data will be processed and stored in accordance with EU General Data Protection Regulation (GDPR) and the Danish Data Protection Act.

Collected data will be stored in the Open Patient Data Exploratory Network (OPEN). OPEN delivers expertise in collection, registration, and documentation of data that meets the rules of protection of personal data. All data will be stored and entered using REDCap® [34, 35].

### Data monitoring

Mortality rates will serve as a safety assessment and will be checked monthly to ensure that mortality is not increased in the intervention group. A data monitoring committee has not been established for this trial as the trial has minimal risk (see Additional file 1).

To ensure adherence to protocol, we will monitor number of biochemistry kits used and logging data from all POCT devices.

### Data analyses

#### Sample size and statistical power

A parallel group design with a 1:1 allocation ratio and a superiority design was chosen for this trial. To our knowledge, the number of patients acutely admitted after a visit from ACNs are unknown. Preliminary results from a pilot-study of the intervention (not yet published) showed that 21% of included participants were acutely admitted after a visit from ACNs. We aimed to detect a clinically significant absolute reduction in acute hospital admission of 10%. Therefore, based on data from the unpublished pilot-study, we aimed in detecting a reduction from 31 to 21% at a significance level 0.05 with a power of 0.80, requiring a total of 602 participants (301 in each arm). Allowing for a 5% dropout after randomisation, 316 in each arm are required (a total of 632 participants).

### Statistical analyses

Statistical analysis will be carried out using STATA® (StataCorp LLC, Texas, USA).

Data analysis will be conducted according to intention to-treat principles and include all eligible participants with available outcome data. Missing data will be reported for each variables, including reasons. Imputation and sensitivity analysis will be used to assess the potential biases caused by missing outcome data.

Descriptive statistics for both groups will be summarised including demographic and baseline characteristics: patient symptoms, referring health professional, Clinical Frailty Scale (CFS) [45, 48], co-morbidities, daily medication, amount of home care, alcohol consumption, smoking, BMI, vital signs (blood pressure, pulse, oxygen saturation, respiratory frequency, and ear temperature), treatment initiations or changes, admissions, re-referrals to the ACNs, contacts to the PCPs, and mortality.

In the intervention group, descriptive statistics will also include POCT measurements and FLUS findings.

Categorical data will be reported as numbers and percentages. Continuous data will be presented as means (SD) on normally distributed data and medians [IQR] with range on non-normally distributed data. Differences in baseline characteristics between the intervention group and control group will be calculated using Chi-squared test, Fischer’s exact test, and Wilcoxon-Mann–Whitney test as appropriate. The statistical significance threshold for all tests is *p* < 0.05.

For the primary outcome univariate and multivariate logistics regression models will be used to investigate risk of admission between groups.

Univariate and multivariate logistic regression models will be used to compare secondary outcomes between the two groups.

Both regression models will be adjusted for possible confounders (gender, age, civil status, co-morbidity, alcohol consumption, smoking, living alone, and BMI).

### Dissemination

The trial outcomes will be disseminated through publications in peer-reviewed journals and presentations at both national and international conferences. Layman-friendly project reports will be distributed to all participants, and comprehensive reports will be forwarded to Kolding Municipality. Social media platforms will be strategically employed to communicate results to a broader audience, encompassing both national and international perspectives.

## Discussion

This study protocol describes the design of an open-label individual randomised controlled trial investigating whether extended use of in-home POCT reduces hospital admissions among older adults. To our knowledge, this is the first individual randomised controlled trial evaluating extended POCT (FLUS and leucocytes with differential count and creatinine) conducted within a community setting. The increasing population of older adults and the rising challenges in the shortage of health professionals necessitate innovative procedures and interventions to maintain the health care system in balance. Through this trial, our aim is to contribute with evidence-based knowledge for the potential development of new interventions beneficial to older adults and hospital administrators. Timely recognition and treatment of acute disease among older adults is central to avoid adverse health outcomes associated with acute hospitalization, such as prolonged hospital stays, readmissions, and declines in functional level [9–11, 49].

### The intervention and primary care physicians

Not all PCPs are familiar with FLUS, but all ACNs will be trained to interpret FLUS findings in relation to clinical examinations and deliver conclusive findings to the PCP.

PCPs routinely use results from venous blood samples (leucocytes with differential count, electrolytes and creatinine). Hence, PCPs have not been instructed on specific cut-offs, relying on their clinical interpretation and knowledge about the patient.

All PCPs in the uptake area are informed about the RCT and its potential effects on the working algorithm during in-home assessments. PCPs, represented in the Advisory Board, have had the opportunity to provide input on the trial’s working algorithm. Thus, we expect that the PCP will adhere to and use results from the intervention in their clinical decision-making.

### Individually randomisation and adherence to protocol

The ACNs will gain competencies and diagnostic abilities to link FLUS findings with clinical findings during the training modules. To ensure that gained competences or awareness of specific diagnoses are not the true intervention, we educate all ACNs and randomise patients individually. By educating all ACNs, we ensure that participants in the control and intervention groups are examined by ACNs with the same level of competences.

We acknowledge potential contamination in the study, specifically protocol violation. ACNs could potentially scan participants or take additional POCT biochemistry on blood if they believe the intervention is better than usual care. However, we have developed several logging systems and checkpoints to ensure adherence to protocol: logging of lung ultrasound, logging of POCT instruments, and checking number of kits used every month.

### Supervision and access to a physician

This trial is the first to investigate whether ACNs can conduct FLUS and extended POCT on blood biochemistry during in-home assessment of older adults. While ACNs have substantial clinical experiences and conduct delegated examinations for PCPs, as FLUS is not widely used in primary care, they will be dependent on supervision for the interpretation of scans by the primary investigator. All contacts with the research team will be registered in the database.

### Over-diagnosing and over-treatment

We hypothesize that extended POCT improves the bedside diagnostic process and qualifies PCPs’ clinical decision-making on treatment, thereby reducing hospital admissions. However, the intervention could lead to an increase in hospital admissions by identifying conditions and diseases requiring further investigations or therapeutic treatment at hospital. We believe that by identifying conditions and diseases earlier, factors such as hospital length of stay, amount of home care, and functional status can be positively influenced during the 30 days follow up, as the patients are admitted prior to functional decline.

Any diagnostic test has false positive results, and both FLUS and bedside blood-analysis have false positive or inconclusive results that can lead to unnecessary additional treatments and hospital admissions.

### Key limitations

Our study is not without limitations. Firstly, the absent of patient-reported outcomes is a notable constraint. However, we plan to address this through forthcoming qualitative research. In-depth interviews with participants, ACNs, and PCPs will be conducted to explore user perspectives and provide insight on how the intervention is experienced. This qualitative approach aims to uncover potential barriers and facilitators affecting the acceptance and use of the intervention.

Secondly, our study is bound by a short-term follow-up period of 30 days. This timeframe may be insufficient to fully capture any long-term effects, particularly concerning outcomes related to functional status and hospitalisation trends. The nuanced influences of various factors beyond this timeframe could affect the comprehensive understanding of the intervention’s sustained effects.

Lastly, the exclusion of participants with moderate to severe cognitive impairment is a recognized limitation. While this exclusion ensures a homogenous study population, it does raise concerns about the generalizability of our findings to a broader population with cognitive challenges. Unfortunately, ethical considerations dictated this exclusion, as outlined in our method section.

Results from this trial will provide essential knowledge on in-home assessments with extended POCT and insight into whether extended POCT can reduce hospital admissions.

### Study status

Ethical approval was given on the 22nd of August 2022 (Project-ID S-20220050) and the study initiated on the 14th of October 2022 and is expected to end the inclusion period on the 31st of August 2023.

The study is registered at clinicaltrials.gov (NCT) with trial registration number: NCT05546073.

### Supplementary Information

**Additional file 1.** The Ethical Commitee’s approved informed consent procedure.

**Additional file 2.** Written information for participants (in Danish).

**Additional file 3.** Conclusive findings on Focused Lung Ultrasound for Acute Community Nurses.

## Data Availability

Not applicable.

## Abbreviations

ACHCS: Acute community health care service
ACN: Acute community nurse
AI: Artificial Intelligence
BMI: Body Mass Index
CFS: Clinical Frailty Scale
CRP: C-reactive Protein
ESCR: Electronic Social Care Record
FLUS: Focused lung ultrasound scan
GDPR: General Data Protection Regulation
OPEN: Open Patient data Explorative Network
PCP: Primary Care Physician
POC: Point-of-care
POCUS: Point-of-care ultrasound
POCT: Point-of-care-technology
VR: Virtual Reality
