# Supplementary material for: Extended use of point-of-care technology versus usual care for in-home assessment by acute community nurses in older adults with signs of potential acute respiratory disease: an open-label randomised controlled trial protocol
Source: BMC Geriatr. 2024 Feb 16;24:161. doi: 10.1186/s12877-024-04774-z (PMC10870485; doi:10.1186/s12877-024-04774-z)
Supplement: Supplementary file 1 — Additional file 1. The Ethical Commitee’s approved informed consent procedure. [file 12877_2024_4774_MOESM1_ESM.docx]

**Appendix 1: The Ethical Commitee’s approved informed consent procedure**

**Hjemmeultralyd af lunge til forebyggelse af akutte hospitalsindlæggelser blandt ældre**

Increased use of point-of-care technology versus usual care for in-home-assessment among older adults for preventing acute hospital admissions: An individual randomised controlled trial

**Ethics**

The ethical principles for medical research as stated by the Declaration of Helsinki, will be applied throughout the studies (1). The trial will follow the CONSORT statements(35). The trial will also be registered and submitted to [www.clinicaltrials.gov](http://www.clinicaltrials.gov).

Participants will receive oral and written information prior to obtaining written consent – for further description see section 1.1.

Participants can withdraw at any time from the study.

- 1. **Informed consent**

Eligible participants for the trial are visited at the eligible participant’s home together by the ACN. The ACN will introduce herself with name and title as acute community nurse. If the eligible participant’s acute situation allows it the eligible participant is informed orally about the study by the ACN. The eligible participant is in his or hers own home, which is a comforting zone for the eligible participant and supporting one’s own will.

The eligible participant will be given time to consider participation in the trial, while the ACN prepares equipment for the usual assessment procedure that have been ordered by the PCP.

After the ACN has prepared equipment, the ACN will ask again whether the eligible participant is willing to participate in the study.

If the patient agrees, a written consent form will be given as well as written information on the trial. The patient is informed that the PCP receives the results

**Lay representative**

All eligible participants have the right to a lay representative. Unfortunately this is not always possible as the study is conducted in the participants own house and due to the fact it is conducted during an acute visit by the ACN. If the potential participant is living together with relatives, they will be encourage to participate in the oral presentation on the study. If no relatives are living together with the potential participant, the potential participant will be encouraged to call a relative by phone. If the eligible participant has no relatives, the ACN will contact a home care nurse who can be lay representative.

**1.2: Incapacitated adults**

Incapacitated adults are often missed and excluded in various studies. Pneumonia and respiratory tract diseases are common in these patients, but difficult to diagnose, as the patients may not describe or express normal symptoms. Diagnosis is therefore delayed. Ultrasound have shown to be a better diagnostic tool than x-ray when it comes to pneumonia among adults with functional decline, and cognitive impairment (19), as the patient can be investigated in bed.
However, we will not include older adults with known moderate to severe cognitive impairment.

**1.3: The Danish Patient Compensation Association**

Participants of the study are covered by the Danish Patient Compensation Association, as the ACNs and primary investigator is an authorized health care personel. The primary investigator is employed through the Geriatric Research Unit at Geriatric Department at Odense University Hospital and collaborate with the emergency department at Kolding Hospital, Sygehus Lillebælt. ACNs are employed at Kolding Municipality.

**1.4: Risks and side effects**

The trial is carried out in a home setting. This ensures a calm environment and thereby less fear and stress, compared to studies conducted in a hospital. The ACNs behave calmly and reassuring that adds to reducing unrest.

Ultrasound is a non-invasive procedure, and consists of acoustic sound energy, not ionizing radiation. The only risk is a potential heating of tissue, but the ultrasound scan used in this study will be performed for a limited time (about 5 minute) by which there is no increased risk of rise in tissue temperature. Moreover, ultrasound is commonly used on patients in the emergency department, as well as by many PCP’s in their offices. Ultrasound is also used in pregnancy screening programmes.

There is no pain attached to ultrasound, but there may be a minor discomfort by having blood samples collected, either by venipuncture for the POCT blood analysis.

**1.5: Benefits and utility value**

The demographic challenge and the reduce of hospital beds require the need for new technologies and organization in the diagnostic work up among acute ill older adults. Acute admissions among older aldults increases the risk of adverse events such as long hospital stays, hospital acquired infections and most importantly decrease in functional status. Many older adults admitted to the hospital wishes they nether had been admitted. Studies shows that treatment in own home provide faster recovery and better rehabilitation, as the older adult are much more mobile in own home, than at the hospital.

The trial is important to conduct, in order to see whether FLUS and POCT on blood samples during in-home assessment, increase the diagnostic precision and early treatment initiation among older adults with symptoms of respiratory infections. If results support our hypothesis, that increased POCT carried out by ACNs reduces hospital admissions, this new intervention holds the potential of upscaling and implementation not only to all municipalities in Denmark, but also worldwide. Other health care systems around the world, even in developing worlds, would be able to train nurses in how to carry out FLUS and increased POCT – thereby providing PCPs with qualified assessments before diagnosis and treatment initiation.

Whatever the outcome, this trial will enhance the competences of ACNs in the municipalities, and the cross- and inter-sectorial collaboration and will serve as a platform for further development of new technologies and working algorithms in the diagnostic work-up among acutely ill older adults. Furthermore, this trial explores the potential of a new working algorithm that supports time-saving in-home clinical diagnostic procedures in a primary care setting, thereby reaching out to vulnerable persons and decreasing inequality in health.
