# Supplementary material for: Extended use of point-of-care technology versus usual care for in-home assessment by acute community nurses in older adults with signs of potential acute respiratory disease: an open-label randomised controlled trial protocol
Source: BMC Geriatr. 2024 Feb 16;24:161. doi: 10.1186/s12877-024-04774-z (PMC10870485; doi:10.1186/s12877-024-04774-z)
Supplement: Supplementary file 2 — Additional file 2. Written information for participants (in Danish). [file 12877_2024_4774_MOESM2_ESM.docx]

**Appendix 2: Written information for participants (in Danish)**

**Hjemme-ultralyd af lunger til forebyggelse af akutte indlæggelser blandt ældre**

**(**Increased use of point-of-care technology versus usual care for in-home-assessment among older adults for preventing acute hospital admissions: An individual randomised controlled trial**)**

**INVITATION**

Vi vil spørge, om du vil deltage i et videnskabeligt forsøg.

Det er frivilligt at deltage i forsøget. Du kan når som helst og uden at give en grund trække dit samtykke tilbage. Det vil ikke for konsekvenser for din videre behandling.

**STED**

Forsøget udføres i dit eget hjem.

**KONTAKTPERSON**

Kontaktperson for projektet er:

Navn: Siri Aas Smedemark

Adresse: Geriatrisk Forskningsenhed

Kløvervænget 10, Indgang 112, 8. sal,

Odense Universitetshospital

E-mail: siri.aas.smedemark@rsyd.dk

Telefon: +45 23 41 56 03

Dato: 15.juni 2022

**FORMÅL**

Forsøget har til formål, at undersøge om en udvidet hjemme-undersøgelses procedure med lunge-ultralydsskanning og ekstra blodprøver udført i eget hjem kan nedsætte antallet af akutte indlæggelser hos ældre.

**PLAN**

Planen for forsøget er, at du tilfældigt udvælges til enten vanlig hjemmeundersøgelses-procedure eller udvidet hjemme-undersøgelses procedure. Vi ved ikke på forhånd hvilken gruppe, du vil komme til at tilhøre, da det er en computer der tilfældigt fordeler deltagere mellem de to procedurer. Først når du takker ja til at deltage, kan vi finde ud af, om du får den vanlige eller den nye hjemmeundersøgelsesprocedure.

Ved *Den vanlige hjemmeundersøgelsesprocedure* vil en akutsygeplejerske måle blodtryk, puls, vejrtræknings-frekvens samt iltmætning i blodet og måle dit infektionstal (CRP) i blodet.

Ved *Den nye hjemmeundersøgelsesprocedure* vil du vil få udført præcis de samme undersøgelser som i den vanlige procedure. I tillæg vil du også få foretaget en lunge-ultralydsskanning og ekstra blodprøver, som vi analyserer på stedet. Den nye procedure tager cirka 20 minutter længere tid end den vanlige procedure.

**BIVIRKNINGER**

Ingen af de ovenstående har bivirkninger, fraset de kendte lette gener ved blodprøvetagning og fornemmelse af kuldskærhed, når ultralydsskanningen foretages.

**DELTAGERINFORMATION**

I den vedlagte deltagerinformation kan du læse mere om, hvad forsøget går ud på, hvad der vil ske med dig, og dine rettigheder, hvis du siger ja.

**Deltagerinformation om deltagelse i et videnskabeligt forsøg for personer med tegn til akut sygdom i eget hjem**

**Hjemme-ultralyd af lunger til forebyggelse af akutte indlæggelser blandt ældre**

**(**Increased use of point-of-care technology versus usual care for in-home-assessment among older adults for preventing acute hospital admissions: An individual randomised controlled trial**)**

Vi vil spørge, om du vil deltage i et videnskabeligt forsøg, der udføres af Geriatrisk Forskningsenhed på Odense Universitetshospital i samarbejde med Lungemedicinsk afdeling, Mikrobiologisk afdeling og Forskningsenheden for Almen Praksis.

Før du beslutter, om du vil deltage i forsøget, skal du fuldt ud forstå, hvad forsøget går ud på, og hvorfor vi gennemfører forsøget. Vi vil derfor bede dig om at læse denne deltagerinformation grundigt.

Du vil få betænkningstid, og du må stille alle de spørgsmål du har om forsøget, inden du beslutter, om du vil deltage.

Hvis du beslutter dig for at deltage i forsøget, vil vi bede dig om at underskrive en samtykkeerklæring.

Det er frivilligt at deltage i forsøget. Du kan når som helst og uden at give en grund trække dit samtykke tilbage. Det vil ikke få konsekvenser for din videre behandling.

**FORMÅL MED FORSØGET**

Det overordnede formål med studiet er at undersøge om vi kan nedsætte antallet af akutte indlæggelse blandt ældre borgere. En af de hyppigste årsager til akut indlæggelse skyldes lungebetændelse og andre akutte sygdomme i lungerne. Ofte kan det være vanskeligt at diagnosticere lungebetændelse blandt ældre, idet ikke alle får feber, fornemmelse af åndenød eller hoste. Nogle gange kan en ændring i ens fysiske formåen være tegn på begyndende sygdom.

Ultralydsskanning af lunger (lunge-UL) kan hurtigt, sikkert og nemt foretages i dit eget hjem, og kan diagnosticere både lungebetændelse og væske på lungerne. Der findes efterhånden mange undersøgelser, der kan laves i eget hjem. Fx kan blodprøver analyseres på stedet, og bidrage til en mere sikker diagnose for, hvorvidt der er tale om en lungebetændelse, eller om tegnene på akut sygdom tyder på anden sygdom end lungesygdom. Lunge-UL er nemt at udføre og bruges i dag også af mange praktiserende læger.

Vi ønsker at undersøge om en nye hjemmeundersøgelsesprocedure med en fokuseret lungeultralydsskanning og ekstra blodprøver udført i eget hjem er bedre til at nedsætte risikoen for en akut indlæggelse ved at sammenligne med den ”vanlige” procedure. Vi har planlagt at undersøge 632 borgere i eget hjem. Du inviteres til at være en af disse.

**PLAN og FREMGANGSMÅDE FOR FORSØGET**

Såfremt du takker ja til at deltage, vil en computer tilfældigt fordele dig til enten at få foretaget den vanlige procedure eller den nye hjemmeundersøgelses procedure.

Ved *Den vanlige hjemmeundersøgelsesprocedure* vil en Akutsygeplejerske måle blodtryk, puls, vejrtræknings-frekvens samt iltmætning i blodet og måle dit infektionstal (CRP) i blodet

*Ved Den nye hjemmeundersøgelsesprocedure* vil du vil få udført præcis de samme undersøgelser som i den vanlige procedure. I tillæg vil du også få foretaget en lunge-ultralydsskanning og ekstra blodprøver, som vi analyserer på stedet. Den nye procedure tager ca 20 minutter længere tid end den vanlige procedure.

Efter endt undersøgelse, hvad enten du har fået den nye eller vanlige hjemmeundersøgelsesprocedure, vil vi informere dig om fundene, og vi kontakter din egen læge for at informere om vores fund. Det er din egen læge, der i fællesskab med dig og med baggrund i vores undersøgelser, beslutter hvad der skal ske videre. Det kan være, der startes antibiotika op på grund af en lungebetændelse, eller du skal have vanddrivende tabletter. Det vil være din egen læge, der følger op på den behandling, der iværksættes.

Hvis din egen læge mener at du skal indlægges, tager din egen læge kontakt til akut modtagelsen på Kolding Sygehus.

**JOURNALOPLYSNINGER OG REGISTRE**

For at få en god forståelse af hvem der har størst glæde af ovennævnte hjemmeundersøgelser, ønsker vi også tilladelse til at se hvilken medicin, du tager og hvilke andre sygdomme du lider af. Det kan vi se hvis du giver tilladelse til, at vi ser dine data i sundhedsregistre, i den elektroniske omsorgsjournal og i din sygehusjournal.

**Sundhedsdatastyrelsen** har registre hvor vi kan se hvilke diagnoser du har, dit medicinforbrug og dine kontakter til lægehus og hjemmeplejen. Ud over dit samtykke, søger vi også tilladelse ved Sundhedsdatastyrelsen. Helt specifikt drejer det sig om Lands Patient Registeret, Lægemiddeldatabasen, Fælles Medicin Kort, Sygesikringsregisteret samt dødsårsagsregisteret.

**Den elektroniske omsorgsjournal** kan vise os hvor meget og hvilken type hjemmehjælp du får, og kan være et mål for hvor skrøbelig du er.

Vi ønsker også at få din tilladelse til at se i din **elektroniske patientjournal**. Vi ønsker at se om du indenfor de sidste 4 uger har været indlagt, og om dette kan være sammenhængende med din aktuelle sygdom. Derudover ønsker vi at se i din elektroniske patient journal 30 dage efter dette besøg for at registrere eventuelle indlæggelser, dit funktionsniveau under indlæggelse, din indlæggelsesårsag, udskrivelsesdiagnose, medicin og registrere eventuelle komplikationer under indlæggelse så som akut forvirringstilstand (også kaldet delir) og hospitalsinfektioner.

Med dit samtykke giver du forsøgsansvarlige, Siri Aas Smedemark, direkte adgang til relevante helbredsoplysninger i din journal, sundhedsregistre samt elektronisk omsorgsjournal, der er nødvendige for at vi kan se, hvem der har størst glæde af hjemmeundersøgelser. Forsøgsansvarlig (Siri Aas Smedemark) søger særskilt tilladelse til at tilgå dine data i sundhedsregistrene samt i din elektroniske omsorgsjournal. Kun forsøgsansvarlig (Siri Aas Smedemark) vil tilgå dine data.

Al indsamlet data vil blive gemt i en sikret database, hvor hver deltager har et unikt deltagernummer. Hverken navn eller adresse bliver gemt sammen med dine helbredsdata.

**BIOLOGISK MATERIALE**

Såfremt du blive fordelt til gruppen der skal have foretaget blodprøver, vil disse blodprøver blive destrueret efter analysearbejdet. Vi vil derfor ikke opbevare dit blod. Det drejer sig kun om 2 blodprøveglas.

**BEHANDLING AF DATA**

Dine data behandles fortroligt og i overensstemmelse med Databeskyttelsesforordningen og Databeskyttelsesloven. Hvis du har spørgsmål til Region Syddanmarks behandling af dine oplysninger, er du altid velkommen til at kontakte Region Syddanmarks databeskyttelsesrådgiver via din digitale postkasse (borger.dk eller e-boks.dk). Du kan også sende en e-mail til databeskyttelsesraadgiver@rsyd.dk. Spørgsmål til projektet bedes rettet til den forsøgsansvarlige, Siri Aas Smedemark.

**NYTTE VED FORSØGET**

Såfremt du bliver fordelt til gruppen der skal have foretaget den nye hjemmeundersøgelsesprocedure, vil du drage nytte af dette projekt, da vi hurtigt kan se på lunge-UL, om du har væske på lungerne eller en lungebetændelse. Derudover vil de ekstra blodprøver også kunne fortælle, om der er tegn til en betændelse i din krop. Hele formålet er, at du skal kunne blive i dit eget hjem, så længe det findes lægeligt forsvarligt. Desuden undgår du transport til sygehus og til din egen læge.

Såfremt du bliver fordelt til gruppen der får foretaget den vanlige hjemmeundersøgelsesprocedure hjælper du os forskere og videnskaben til at kunne afklare om den nye hjemmeundersøgelsesprocedure virkelig kan nedsætte antallet af akutte hospitalsindlæggelser. På den måde bidrager du i det store billede til at vi kan se om den nye hjemmeundersøgelsesprocedurer har effekt på akutte indlæggelse.

Hvis vores forsøg viser, at vi hurtigt og effektivt kan iværksætte korrekt behandling med disse hurtige undersøgelser, andre ældre borgere i Danmark få nytte af dette forsøg. Forsøget vil kunne udvides til alle kommuner, så mange flere ældre borgere kan få gavn af undersøgelserne.

**Bivirkninger, risici, komplikationer og ulemper**

Blodprøver:

- Kan give let smerte idet nålen skal gennem huden
- Det kan give midlertidige blodudtrækninger, der hvor blodprøven er taget
- Der er en meget lille risiko for infektion omkring indstiksstedet, men det er meget sjældent. Hvis du oplever det, må du endelig kontakte os, så vi kan få det behandlet og registreret.

Ultralydsskanning:

- Der smøres en gel på din overkrop, som kan føles kølig og let klistrende.
- Der er *ikke* stråle-risiko ved ultralyd, da det drejer sig om lydbølger.

Der kan være risici ved forsøget, som vi endnu ikke kender. Vi beder dig derfor om at fortælle, hvis du oplever problemer med dit helbred, mens forsøget står på. Hvis vi opdager bivirkninger, som vi ikke allerede har fortalt dig om, vil du naturligvis blive orienteret med det samme, og du vil skulle tage stilling til, om du ønsker at fortsætte i forsøget.

**UDELUKKELSE FRA OG AFBRYDELSE AF FORSØGET**

Du vil altid kunne trække dit samtykke tilbage og udgå af studiet.

I tilfælde af akut situation under forsøget kan vi være nødsaget til at afbryde forsøget – eksempelvis såfremt du bliver ukontaktbar.

**OPLYSNINGER OM ØKONOMISKE FORHOLD:**

Vi har modtaget støtte fra:

- Region Syddanmarks ph.d.pulje: 518.000 kr. til ph.d.-løn
- Syddansk Universitet: 580.500 kr. til ph.d.-løn
- Gangstedfonden: 500.000 kr. til ph.d.-løn
- Geriatrisk Afdeling og Medicinsk Afdeling på Kolding Sygehus: 161.000 kr. til ph.d.-løn
- OUH’s Innovationsfond: 150.000 kr. til indkøb af udstyr til projektet
- Gerikuffert-projektet: udlån af udstyr til værdi af 140.000 kr.
- Hartmann fonden: 100.000 kr. til indkøb af udstyr til projektet
- A. P. Møller Fondens Lægefonden: 55.000 kr. til indkøb af udstyr til projektet.
- Grosserer L. F. Foghts fond: 100.000 kr. til indkøb af udstyr til projektet
- Karen Elise Jensens fond: 1.000.000 kr. til udstyr og drift samt projektsygeplejerske

Hverken den ph.d.-studerende eller vejledere på projektet har økonomisk tilknytning til støttegivere.

Professor Karen Andersen-Ranberg samt ph.d.-studerende Siri Aas Smedemark er begge initiativtagere til studiet.

**ADGANG TIL FORSØGSRESULTATER**

Selve studiet forventes at være afsluttet når 632 borgere er blevet inkluderet i projektet. Dette forventer vi er opnået maj 2023. Offentliggørelsen af resultater fra studiet forventes at blive i efteråret 2023.

De anonyme resultater fra studiet vil blive offentliggjort i medicinske journaler og tidsskifter. Derudover vil der blive udfærdiget rapporter til Kolding Kommune og de praktiserende læger.

Vi håber, at du med denne information har fået tilstrækkeligt indblik i, hvad det vil sige at deltage i forsøget, og at du føler dig rustet til at tage beslutningen om din eventuelle deltagelse. Vi beder dig også om at læse det vedlagte materiale ”Forsøgspersonens rettigheder i et sundhedsvidenskabeligt forskningsprojekt”.

Hvis du vil vide mere om forsøget, er du meget velkommen til at kontakte

*Siri Aas Smedemark, MD, læge, Ph.d.-studerende*

Geriatrisk Forskningsenhed,

Odense Universitetshospital

Kløvervænget 10, Indgang 112, 8. sal,

5000 Odense C

+45 23 41 56 03

[siri.aas.smedemark@rsyd.dk](mailto:siri.aas.smedemark@rsyd.dk)

Med venlig hilsen

Siri Aas Smedemark
