# Supplementary material for: Extended use of point-of-care technology versus usual care for in-home assessment by acute community nurses in older adults with signs of potential acute respiratory disease: an open-label randomised controlled trial protocol
Source: BMC Geriatr. 2024 Feb 16;24:161. doi: 10.1186/s12877-024-04774-z (PMC10870485; doi:10.1186/s12877-024-04774-z)
Supplement: Supplementary file 3 — Additional file 3. Conclusive findings on Focused Lung Ultrasound for Acute Community Nurses. [file 12877_2024_4774_MOESM3_ESM.docx]

| **Appendix** **3**: **Conclusive findings on Focused Lung Ultrasound for Acute Community Nurses** | | |
| --- | --- | --- |
| **No. Zones scanned** | 8 / 14 | |
| **Are there lung-slinding?** | YES | NO |
| - If no:   - Lung-point?   - Pneumothorax? | YES / NO  YES / NO | |
| **Any B-lines?** | YES | NO |
| - If yes:   - Interstitiel syndrome? |  |  |
| **Are pleural effusion present?** | YES | NO |
| - If yes:   - Characteristics:   - Size: | Simple / complex  Small / moderat / large | |
| **Any consolidated lung?** | YES | NO |
| - If yes | Pneumonia  Possible pneumonia  Not pneumonia  Other, describe: | |
| **Any other pathology?**   - If yes:   - Describe |  | |
| **Are their signs of pneumonia?** | YES | NO |
| - If yes, what signs are you basing this on? | Multiple focal B-lines  Lung consolidations  Irregular pleura line  Irregular pleura line and focal B-lines  Other, describe: | |
| **How would you rate the quality of your scans from 1-5?** | 1 Extremely low quality  2 Low quality  3 Acceptable quality  4 Good quality  5 Perfect quality | |
| **How certain are you on your findings from 0-100?** | Not certain Tolerable Certain   1. 50 100 | |
